# Comparing patterns of recent mental health service use for predicting suicidal events following emergency department mental health visits in the United States: A national cohort study

**DOI:** 10.1007/s00127-025-03016-w

**Published:** 2025-12-02

**Authors:** Timothy Schmutte, Steven C. Marcus, Ming Xie, Sara Wiesel Cullen, Tony Liu, Lyle H. Ungar, Nick Cardamone, Nathaniel J. Williams, Mark Olfson

**Affiliations:** 1 Department of Psychiatry, Yale University School of Medicine, 300 George Street, Suite 901, New Haven, CT US; 2 School of Social Policy & Practice, University of Pennsylvania, Philadelphia, PA US; 3 Department of Psychiatry,Perelman School of Medicine, University of Pennsylvania, Philadelphia, PA US; 4 Department of Computer Science, Mount Holyoke College, South Hadley, MA US; 5 Department of Computer and Information Science, University of Pennsylvania, Philadelphia, PA US; 6 School of Social Work, Boise State University, Boise, ID US; 7 Department of Psychiatry,Vagelos College of Physicians and Surgeons, Columbia University and the New York State Psychiatric Institute, New York, NY US

**Keywords:** Emergency Department, Mental Health, Suicide, Self-Harm, Health Services, Healthcare Utilization

## Abstract

**Objective:**

To examine patterns of recent service use to predict non-fatal suicidal events shortly following emergency department (ED) visits for mental health.

**Methods:**

For this retrospective cohort study, we used Optum electronic health record data from 2,445,597 ED mental health episodes (2015–2022) for persons aged *≥* 10 years. We then constructed a series of logistic regression models to evaluate how six permutations of characterizing prior 180-day mental health service use predicted acute non-fatal suicidal events within 180 days of ED discharge beyond demographic characteristics and ED mental health diagnoses. Model performance was assessed by area under the receiver operating curve (AUC).

**Results:**

Overall, 7.2% (*n* = 176,000) of episodes resulted in an acute suicidal event within 180 days. Model performance improved from demographic characteristics and ED mental health diagnoses (AUC = 0.76) when past 180-day service use variables were added, but with minimal differences between a binary variable of any mental health service use compared to monthly counts, weighted slopes, or interactions (AUC’s 0.78–0.82). The final model containing demographics, ED mental diagnosis, and past 180-day service use yielded an AUC of 0.83. The most predictive past service use variables were any inpatient or ED event for self-harm or suicide ideation (OR = 5.45, 95% CI = 5.37, 5.54) and any ED mental health visit (OR = 1.87, 95% CI = 1.84, 1.90).

**Conclusions:**

As part of evaluating suicide risk in ED settings, information about recent acute care for mental health, suicidal ideation, or self-harm use significantly contributes to the short-term prediction of non-fatal suicidal events.

## Introduction

Suicide risk is critically high in the months following emergency department (ED) visits for mental health problems, especially among ED patients who were treated for suicidal ideation or self-harm [1, 2]. However, accurately identifying high risk ED patients remains a vexing challenge as screening by itself does not reduce risk of future suicidal behavior[3] and 33%−50% of patients denied suicidal ideation prior to their non-fatal or fatal suicide attempt [4]. In an effort to improve identification of high suicide risk patients, various modelling techniques using health care data have been recently applied, including machine learning approaches[5], trajectory-based modelling of service use[6], and latent class models [7]. 

Deeper examination of patterns of prior health care encounters may provide valuable insights as growing research suggests that suicide risk is time-varying and may be associated with health care use. For example, one study found that a concentration of mental health service use in a 3-month period prior to an index visit was significantly more predictive than longer lookback periods of non-fatal and fatal self-harm within 90 days [8]. Other research suggests that repeated or escalating inpatient or ED mental health episodes may indicate unresolved crises, whereas individuals with erratic, declining, or missed outpatient mental health visits may be particularly at-risk [9–13]. 

Advanced modelling techniques using health care data to predict suicide-related outcomes are superior to traditional clinical approaches [14]. Most research using health records has focused on identifying at-risk patients following outpatient visits or hospitalizations [15] or among relatively narrow ED populations. For example, prior prediction models focused on suicide-related outcomes following ED encounters include patients treated for self-harm[16], aged *≤*18 years[17], or who completed a suicide ideation rating scale during the ED encounter [18]. In a recent study using health records to predict suicide mortality within 90 days of an ED encounter for any mental health condition, the model yielded an area under the receiver operating curve (AUC) of 0.82 (95% CI = 0.81–0.84)[19]. In this study, multiple approaches were used to characterize prior service use, including the total number of mental health encounters in outpatient, ED, and inpatient settings as well as 52 possible time patterns reflecting differing permutations (e.g., first onset, most recent occurrence, and increase or decrease over time) [19]. 

In most suicide prediction models, static characteristics including demographic (i.e., male sex, race, and age) and diagnostic features (e.g., major depression and psychosis) are salient suicide risk factors with prior treatment for deliberate self-harm being the most robust clinical predictor [15]. Recent developments suggest that adding dynamic, time-sensitive changes in healthcare utilization patterns (e.g., visit frequency and timing) can improve the predictive performance above and beyond demographic and clinical characteristics [20, 21]. However, it is unclear which indicators of prior service use are most effective at capturing complex temporal relationships (e.g., slope-related trends and other non-linear patterns of service use preceding an index mental health visit) to enhance the prediction of suicide-related outcomes. In this brief report, we evaluate the incremental performance of different ways of characterizing recent mental health care use, beyond demographic and diagnostic characteristics, in predicting acute suicidal events within 180 days of an ED mental health visit as part of the development of a clinical decision support tool for ED physicians to assess suicide risk. Based on emerging research that incorporating temporality of recent service use improves model performance[20, 21], we hypothesized that more granular approaches (e.g., increased clustering of escalating encounters more proximal to the time of a non-fatal suicidal event) would provide more accurate predictions of subsequent suicide risk.

## Methods

### Data sources

This retrospective cohort study used de-identified, structured electronic health record (EHR) data from the Optum Labs Data Warehouse (OLDW). The Optum EHR database contains longitudinal health information for over 200 million commercial, Medicaid, and Medicare Advantage enrollees representing a range of ages and geographical regions. The database includes diagnoses, procedures, medications, laboratory results, hospitalizations, and outpatient visits from integrated delivery networks including > 140,000 providers from > 700 hospitals and > 7,000 clinics. For the current study, EHR data were limited to ED visits between 9/30/2015-9/30/2022, which coincides with the adoption of ICD-10 in U.S. healthcare settings. In the U.S., ICD diagnostic codes are typically entered for billing and clinical documentation purposes. Because codes are often required to justify payment for services rendered, diagnoses that are connected to billable encounters tend to be more consistently and accurately coded. Research in the U.S. demonstrated that coding for self-harm in ED settings are well-captured (i.e., 88%) and improved with the transition in 2015 from ICD-9 to ICD-10[22, 23].

The data for this study, which are deidentified and previously collected, were deemed exempt from human participants review by the University Institutional Review Board. According to U.S. federal regulations governing human subjects research, secondary research utilizing data that is recorded anonymously and cannot be linked to subjects through identifiers is classified as exempt under 45 CFR 46.

### Cohort assembly

We identified all ED visits persons aged > 10 years containing an EHR diagnosis for mental disorder (ICD-9 codes 290–319; ICD-10 codes F01-F99), intentional self-harm (ICD-9 codes E950-959; ICD-10 codes X71–X83), poisoning by drugs, medications or biological substances coded as intentional self-harm (ICD-10 codes T36 – T50), toxic effects of nonmedical substances coded as intentional self-harm (ICD-10 codes T51 – T65) asphyxiation, suffocation, or hanging codes as intentional self-harm (ICD-10 code T71), suicide attempt (ICD-10 code T14.91) or suicidal ideation (ICD-9 code V62.84; ICD-10 code R45.851).

We excluded ED visits for which the only mental health diagnosis was nicotine dependence (ICD-9 305.1; ICD-10 F17). Study visits were also excluded for contiguous observation episode prior to the index ED visit, length of ED stay of >4 days, and no evidence of healthcare activity within the participating health networks in the 180 days prior to the index visit. We selected a length of stay of less than 4 days to focus on patients receiving acute ED care that reflect individuals who undergo assessment, stabilization, and disposition (either discharge or admission) within the standard ED workflow, rather than patients with extended boarding in the ED. Individuals with no evidence of healthcare activity in the 180 days prior to their index ED episode or in the 180 post-index period were excluded because the absence of data compromised our ability to discern whether they truly had no care or were treated in a different health system not included in the Optum database. Episodes occurring within 2 days of discharge were combined as they were considered a continuation of care from the initial event based on prior research [24]. All ED episodes meeting these criteria were included in analyses, thus individual patients could contribute more than one ED mental health encounter. The final sample size was 2,445,597 ED episodes, representing 1,341,253 individuals.

### Outcome

Acute non-fatal suicidal events were defined as any ED or inpatient claim within 180 days of ED discharge with a diagnosis of intentional self-harm (ICD-9 codes E950-959; ICD-10 codes X71–X83), poisoning by drugs, medications or biological substances coded as intentional self-harm (ICD-10 codes T36 – T50), toxic effects of nonmedical substances coded as intentional self-harm (ICD-10 codes T51 – T65) asphyxiation, suffocation, or hanging codes as intentional self-harm (ICD-10 code T71), suicide attempt (ICD-10 code T14.91), or suicidal ideation (ICD-9 code V62.84; ICD-10 code R45.851) [25]. 

### Predictors

We selected patient demographic characteristics (sex, race/ethnicity, and age) and mental health diagnoses during the ED encounter as well as three binary characterizations of past 180-day service use: (1) any prior ED visit for any mental health disorder, self-harm, or suicide ideation; (2) any prior inpatient or ED event for self-harm or suicide ideation; and (3) any prior service use for self-harm or suicide ideation in any setting (i.e., ED, inpatient, outpatient). In addition to this predictor group, six characterizations of prior service use were selected to represent progressively more granular approaches that account for temporality and intensity of healthcare use as research suggests that this approach enhances predictive modelling of subsequent suicide risk [20, 21]. 

Each of the six modelling strategies was designed to progressively test how temporal patterns of past 180-day service use predicted non-fatal suicidal events, beginning with aggregate measures and moving toward increasingly detailed temporal configurations. The first modelling approach included a single binary indicator for any mental health-related encounter in any setting (i.e., ED, inpatient, outpatient) 180 days prior to the index ED event. The second model included a single independent variable containing the aggregate count of mental health encounters in any setting in the prior 180 days. The third approach was a set of six predictors representing the monthly count of mental health encounters in any setting for each of the six months in the prior 180 days. The fourth modelling strategy was a weighted slope based on the sum of the six monthly counts with each count multiplied by a monthly exponential decay function (see footnote in Table 2). The fifth approach included a set of six binary indicators of any mental health event in any setting in each month during the prior 180 days. The sixth modelling approach included a set of factors containing the interaction of the six monthly binary service use indicators from the prior model (64 combinations) to provide all permutations of monthly service use patterns. This operationalization accounted for fine-grained temporal variations in past service use (e.g., consistent, intermittent, or front- or back-loaded service engagement) to assess whether these distinct trajectories were differentially associated with the outcome.

### Analysis

The analysis was conducted in 3 stages. First, we characterized the ED cohort and the sample who experienced an acute non-fatal suicidal event following ED discharge based on demographic, diagnostic, and prior mental health service use characteristics using unadjusted logistic regression. Second, we constructed a series of logistic regression models with different predictor set combinations to evaluate how different methods of characterizing mental health service use patterns in the prior 180 days influenced the AUC above and beyond characteristics from the ED encounter. Our base model utilized only demographic characteristics. The second model included demographic and mental health diagnosis variables from the ED encounter. Each subsequent model included demographic and diagnosis features from the ED encounter as well as 1 of the 6 permutations to characterizing monthly mental health service use patterns in the 180 days prior to the ED episodes described in the previous section. Using this approach, separate logistic regression analyses evaluated the relative strength of three characterizations of prior 180-day mental health service use: (1) prior ED visit for any mental health disorder, self-harm, or suicide ideation; (2) prior inpatient or ED event for self-harm or suicide ideation; and (3) any prior service use for self-harm or suicide ideation. To ensure the robustness of our findings, we used a 2/3 training set and 1/3 test set split to generate and evaluate all models and ensure that the performance metrics were reflective of the model’s generalizability. Each model was assessed by examining the out-of-sample test AUC, a measure of model performance, to evaluate how well the model distinguished between individuals who did and did not experience acute non-fatal suicidal events within 180 days of an ED visit.

## Results

### Cohort characteristics

Among the 2,445,597 ED episodes, most were by patients who were women (58.1%), white non-Hispanic (70.5%), either 18–34 or 45–64 years old (29.9% and 30.9%, respectively), and diagnosed with either depressive (31.9%) or anxiety (40.4%) disorders (Table 1). Overall, 7.2% (*n* = 176,000) resulted in an acute non-fatal suicidal event within 180 days. Although female and white, non-Hispanic patients made up most of the ED episodes, in unadjusted models the demographic characteristics associated with significantly higher odds of an acute suicidal event following ED discharge were being male (OR = 1.82) or Black non-Hispanic (OR = 1.25). Lower likelihood of an acute suicidal event within 180 days were associated with being aged *≥* 65 years and diagnosed with anxiety disorders during the ED visit. Clinical characteristics associated with increased likelihood of an acute suicidal event included an ED diagnosis of suicidal ideation or self-harm (OR = 6.22), personality disorder (OR = 4.49), psychotic disorder (OR = 3.86), or bipolar disorder (OR = 2.21).

**Table 1:** Characteristics of emergency department visits with mental health diagnoses

	% of total sample *N* = 2,445,597	% with acute suicidal event within 180 days *N* = 176,000	Unadj OR
*Sex*	%	%	
Male	41.9	9.6	1.82(1.80,1.84)
Female	58.1	5.5	ref
*Race/Ethnicity*			
Hispanic	6.7	6.7	0.96(0.94,0.98)
Black, non-Hispanic	18.3	8.5	1.25(1.23,1.26)
White, non-Hispanic	70.5	6.9	ref
Asian, Non-Hispanic	0.6	4.6	0.65(0.60,0.71)
Other	3.9	7.1	1.03(1.00,1.06)
*Age (years)*			
10–17 years	5.7	9.8	1.16(1.14,1.19)
18–34 years	29.9	8.5	ref
35–44 years	17.8	8.7	1.03(1.02,1.04)
45–64 years	30.9	7.4	0.86(0.85,0.87)
65 + years	15.8	1.6	0.18(0.17,0.18)
*ED Mental Health Conditions*			
Suicidal ideation or self-harm			
Yes	8.0	26.6	6.22(6.14,6.29)
No	92.0	5.5	ref
Psychotic disorders			
Yes	7.4	20.2	3.86(3.81,3.91)
No	92.6	6.2	ref
Depressive disorders			
Yes	31.9	8.4	1.28(1.27,1.29)
No	68.1	6.7	ref
Bipolar disorders			
Yes	9.8	13.4	2.21(2.18,2.24)
No	90.2	6.5	ref
Anxiety disorders			
Yes	40.4	5.4	0.63(0.62,0.64)
No	59.6	8.4	ref
Trauma- and stress-related disorders			
Yes	6.3	12.2	1.89(1.86,1.92)
No	93.7	6.9	ref
Disruptive behavior disorders			
Yes	1.1	17.3	2.75(2.67,2.84)
No	98.9	7.1	ref
Personality disorders			
Yes	1.4	25.1	4.49(4.38,4.60)
No	98.6	6.9	ref
Nicotine-related disorders *			
Yes	29.3	9.8	1.66(1.64,1.67)
No	70.7	6.1	ref
Alcohol-related disorders			
Yes	14.7	11.2	1.80(1.78,1.82)
No	85.3	6.5	ref
Other substance related disorders			
Yes	14.6	10.4	1.63(1.61,1.65)
No	85.4	6.7	Ref
*180-Day Prior Service Use*			
ED visit for mental health, self-harm, or suicide ideation			
Yes	40.0	12.9	4.24(4.20,4.29)
No	60.0	3.4	ref
Prior inpatient or ED event for self-harm of suicide ideation			
Yes	8.1	39.6	14.5(14.3,14.6)
No	91.9	4.3	ref
Any prior service use for self-harm or suicide ideation			
Yes	62.4	9.7	3.37(3.32,3.41)
No	37.6	3.1	ref
Summary of 3 prior service use variables			
Yes	69.8	9.2	3.91(3.85,3.97)
No	30.2	2.5	ref

* Nicotine-related disorders were not a qualifying diagnosis and included only if comorbid with another mental health condition

### Examining different predictor models of acute suicidal events

The base logistic regression models containing only demographic characteristics had an AUC of 0.63, which increased after adding mental health diagnoses from the ED encounters to an AUC to 0.76 (Table 2). For all three mental health service variables, the addition of prior 180-day mental health service use permutations to the model only modestly increased AUC values, however, there was little improvement beyond the binary service use variable for any event in the prior 180 days (e.g., AUC values ranging from 0.78 to 0.82) with more nuanced permutations of prior service use (e.g., monthly counts, weighted slopes, interactions) failing to yield meaningful increases in model performance (Table 2).

**Table 2:** Comparison of areas of under the curve (AUCs) for 180-day prior service use variables predictive of acute non-fatal suicidal events within 180 days following emergency department mental health encounter

	Prior ED visit for mental health, self-harm, or suicide ideation	Prior inpatient or ED event for self-harm or suicide ideation	Any prior service use for self-harm or suicide ideation
Characterizing ED visit only			
Demographics only	0.634	0.634	0.634
Demographics and diagnosis from ED visit	0.760	0.760	0.760
Characterizing prior 180 days service use *			
1. Any event in prior 6 months (1 predictor)	0.795	0.817	0.783
2. Count of events in prior 6 months (1 predictor)	0.801	0.815	0.794
3. Monthly counts of events in each of prior 6 months (6 predictors)	0.802	0.815	0.795
4. Weighted slope of each person’s monthly use** (6 predictors)	0.802	0.815	0.795
5. Monthly presence of any event in prior 6 months (6 predictors)	0.806	0.819	0.797
6. Interactions of monthly binary (32 predictors)	0.806	0.819	0.798

* Models included demographic characteristics and diagnosis from ED visit

** Weighted slope based on monthly exponential decay function of time prior to ED episode multiplied by monthly event count (1 month = 1.00, 2 months = 0.50, 3 months = 0.250, 4 months = 0.125, 5 months = 0.063, 6 months = 0.031)

When demographic characteristics, ED mental health diagnosis, and three characterizations of prior 180-day service use variables were used together in a logistic model to predict acute suicidal events within 180 days of the ED encounter, the model achieved an AUC value of 0.83. The strongest predictor was prior inpatient or ED event for self-harm or suicide ideation (OR = 5.45, 95% CI = 5.37, 5.54) followed by prior ED visit for any mental health disorder, self-harm, or suicide ideation (OR = 1.87, 95% CI = 1.84, 1.90) and any prior service use for self-harm or suicide ideation (OR = 1.56, 95% CI = 1.53, 1.59).

## Discussion

This retrospective cohort study examined factors associated with suicidal and self-harm events within 180 days of an ED mental health visit with a focus on comparing the incremental predictive performance of different permutations of prior service use. Key findings include that suicidal ideation or self-harm, personality disorder, psychotic disorder, or bipolar disorder diagnosed during the ED encounter were clinically meaningful predictors of non-fatal suicidal events within 180 days. Our final prediction model using demographic, ED diagnoses, and prior 180-day service variables had an overall AUC value of 0.83 for non-fatal suicidal events. Contrary to our hypothesis, more granular modelling approaches of prior service use that accounted for temporality and intensity of healthcare encounters (e.g., monthly counts, weighted slopes, interactions) did not meaningfully improve predictive performance beyond a simple binary indicator for any mental health-related encounter in the prior 180 days.

The classification performance of our final model compares favorably with previous studies using structured EHR data to predict non-fatal and fatal suicide-related events shortly following ED mental health visits, which observed AUC values ranging from 0.65–0.82[17–19]. Whereas some studies that examined different temporal patterns of prior service use found that binary indicators to be as predictive of suicide-related outcomes as more sophisticated representations[19, 26], other studies found temporally-informed service use predictors to be superior [20, 27]. Similarly, one study observed health care encounters in the 3 months prior to a mental health visit were significantly more predictive than more distant encounters of non-fatal and fatal self-harm within 90 days [28]. One potential reason for the inconsistent results may include differences in patient populations as some have focused on ED and inpatient patients[19, 26], whereas others included outpatients or all patients in a healthcare system [20, 27, 28]. Additional research is needed to inform how patients with elevated suicide risk can be identified using predictive modelling approaches.

### Implications

From a clinical perspective, these findings can assist ED clinicians in screening patients to identify those who carry the highest short-term risk of subsequent non-fatal suicidal events and most in need of more personalized, stepped-up care. Single encounter, in-person suicide prevention interventions significantly improve linkage with follow-up mental health care and reduce subsequent suicide attempts [29]. Oher brief interventions show promise in reducing suicide risk, such as caring contacts (e.g., texts, post cards), follow-up case management and supportive counseling, and safety planning [3, 30, 31]. 

In the U.S., the Joint Commission requires suicide screening of all patients who are being evaluated or treated for behavioral health conditions as their primary reason for care using a validated screening tool, such as the Columbia Suicide Severity Rating Scale (C-SSRS) [32]. Our final model (AUC = 0.83) performed substantially better than the reported value for screening with the C-SSRS for predicting suicide attempt (AUC = 0.59) following an ED episode [33]. Given that many patients deny suicidal ideation prior to self-harm [4] and barriers to disclosure during ED assessment of suicide risk have been identified[34, 35], inquiries about prior mental health service use may be a viable clinical strategy to reduce stigma and discomfort to better inform risk assessment. Especially in healthcare settings that do not routinely screen, EHR information about prior acute mental health care can assist clinicians with stratifying risk and allocating limited clinical resources based on patient need. Additionally, 53.7% of U.S. community hospitals lack psychiatric services for ED consultation[36], requiring frontline clinicians to make difficult decisions regarding risk assessment and appropriate care that could be informed by reviewing a patient’s EHR for prior acute psychiatric care. Because of its predictive value, information about past psychiatric hospitalization has been incorporated into secondary screening tools to stratify suicide risk in ED settings [37, 38]. 

### Limitations

This study has several limitations. First, our cohort consisted of ED patients treated for mental health disorders and is not representative of the general population. Although this dataset included a diverse range of publicly insured, commercially insured, and uninsured patients, roughly 20% of adults who attempt suicide do not have mental disorders [39] and thus may not be represented in this ED mental health sample. Second, we relied on ICD diagnosis codes and EHR diagnoses, which were based on routine clinician assessments and were not subjected to independent expert validation. Some concerns exist over the validity and completeness of ICD codes for suicidal ideation and behaviors[40, 41], however recent U.S. research demonstrated significant improvements in completeness and accuracy of coding of intentional self-injury and self-poisoning with the transition from ICD-9 to ICD-10 in the U.S. on October 1, 2015[22, 23]. Additionally, our findings are limited to episodes of acute suicidal ideation or self-harm that resulted in an ED encounter and may not generalize suicide-related events that resolved in the community or were treated in outpatient settings. Third, because of our reliance on ICD codes, we were unable to distinguish suicide attempts from non-suicidal self-injury which can represent distinct clinical entities and confer different risk of subsequent self-harm [42, 43]. Additionally, ICD codes do not provide details about the clinical severity or context of deliberate self-harm.

## Conclusions

ED clinicians commonly face clinical uncertainties in evaluating suicide risk, even when using standardized screening measures. In the current study, a prediction model using structured EHR data demonstrated relatively accurate prediction of acute non-fatal suicidal events within 180 days following ED mental health encounters. As part of evaluating suicide risk in ED settings, these findings can help to inform clinical determinations for which patients warrant higher levels of follow-up care.

## Data Availability

No datasets were generated or analysed during the current study.

## References

[1] Ahmedani BK, Westphal J, Autio K et al (2019) Variation in patterns of health care before suicide: a population case-control study. Prev Med. Oct;127:105796. 10.1016/j.ypmed.2019.105796 31400374 10.1016/j.ypmed.2019.105796 PMC6744956

[2] Olfson M, Gao YN, Xie M, Wiesel Cullen S, Marcus SC (2021) Suicide risk among adults with mental health emergency department visits with and without suicidal symptoms. J Clin Psychiatry Oct 26(6). 10.4088/JCP.20m13833 10.4088/JCP.20m13833 PMC8672323 34705348

[3] Miller IW, Camargo CA Jr, Arias SA et al (2017) Suicide prevention in an emergency department population: the ED-SAFE study. JAMA Psychiatr 74(6):563–570. 10.1001/jamapsychiatry.2017.0678 10.1001/jamapsychiatry.2017.0678 PMC5539839 28456130

[4] Obegi JH (2021) How common is recent denial of suicidal ideation among ideators, attempters, and suicide decedents? A literature review. Gen Hosp Psychiatry 72:92–95. 10.1016/j.genhosppsych.2021.07.009 34358807 10.1016/j.genhosppsych.2021.07.009

[5] Boudreaux ED, Rundensteiner E, Liu F et al (2021) Applying machine learning approaches to suicide prediction using healthcare data: overview and future directions. Front Psychiatry. 10.3389/fpsyt.2021.707916 34413800 10.3389/fpsyt.2021.707916 PMC8369059

[6] Chitty KM, Sperandei S, Carter GL et al (2023) Five healthcare trajectories in the year before suicide and what they tell us about opportunities for prevention: a population-level case series study. eClinicalMedicine. 10.1016/j.eclinm.2023.102165 37649805 10.1016/j.eclinm.2023.102165 PMC10462847

[7] Ahmedani BK, Cannella CE, Yeh H-H et al (2022) Detecting and distinguishing indicators of risk for suicide using clinical records. Translational Psychiatry 12(1):1–9 35831289 10.1038/s41398-022-02051-4 PMC9279332

[8] Wolock CJ, Williamson BD, Shortreed SM et al (2024) Importance of variables from different time frames for predicting self-harm using health system data. J Biomed Inform 160:104750. 10.1016/j.jbi.2024.104750 39557209 10.1016/j.jbi.2024.104750 PMC11891787

[9] Tang S, Reily NM, Arena AF et al (2022) People who die by suicide without receiving mental health services: A systematic review. systematic review. Frontiers Public Health 2022-January- 18:9. 10.3389/fpubh.2021.736948 10.3389/fpubh.2021.736948 PMC8804173 35118036

[10] Schaffer AL, Buckley NA, Schumann J et al (2024) Patterns of medicine use in the year prior to death by suicide: an Australian population-based case series study. eClin Med. 10.1016/j.eclinm.2024.102858 10.1016/j.eclinm.2024.102858 PMC11474424 39416392

[11] Renaud J, Séguin M, Lesage AD, Marquette C, Choo B, Turecki G (2014) Service use and unmet needs in youth suicide: a study of trajectories. Can J Psychiatry 59(10):523–530. 10.1177/070674371405901005 25565685 10.1177/070674371405901005 PMC4197786

[12] Myhre MØ, Walby FA, Bramness JG, Mehlum L (2024) Trajectories of service contact before suicide in people with substance use disorders—a national register study. Arch Suicide Res 28(1):200–215. 10.1080/13811118.2022.2151959 36472383 10.1080/13811118.2022.2151959

[13] King CA, Brent D, Grupp-Phelan J et al Five profiles of adolescents at elevated risk for suicide attempts: differences in mental health service use. J Am Acad Child Adolesc Psychiatry. 2019/12/09/ 2019;doi:10.1016/j.jaac.2019.10.015 10.1016/j.jaac.2019.10.015 PMC7280071 31830523

[14] Schafer KM, Kennedy G, Gallyer A, Resnik P (2021) A direct comparison of theory-driven and machine learning prediction of suicide: a meta-analysis. PLoS One 16(4):e0249833. 10.1371/journal.pone.0249833 33844698 10.1371/journal.pone.0249833 PMC8041204

[15] Pigoni A, Delvecchio G, Turtulici N et al (2024) Machine learning and the prediction of suicide in psychiatric populations: a systematic review. Transl Psychiatry 14(1):140. 10.1038/s41398-024-02852-9 38461283 10.1038/s41398-024-02852-9 PMC10925059

[16] Sanderson M, Bulloch AGM, Wang J, Williams KG, Williamson T, Patten SB (2020) Predicting death by suicide following an emergency department visit for parasuicide with administrative health care system data and machine learning. EClinicalMedicine 20:100281. 10.1016/j.eclinm.2020.100281 32300738 10.1016/j.eclinm.2020.100281 PMC7152812

[17] Haroz EE, Kitchen C, Nestadt PS, Wilcox HC, DeVylder JE, Kharrazi H (2021) Comparing the predictive value of screening to the use of electronic health record data for detecting future suicidal thoughts and behavior in an urban pediatric emergency department: a preliminary analysis. Suicide Life Threat Behav 51(6):1189–1202. 10.1111/sltb.12800 34515351 10.1111/sltb.12800 PMC8961462

[18] Nock MK, Millner AJ, Ross EL et al (2022) Prediction of suicide attempts using clinician assessment, patient self-report, and electronic health records. JAMA Netw Open 5(1):e2144373–e2144373. 10.1001/jamanetworkopen.2021.44373 35084483 10.1001/jamanetworkopen.2021.44373 PMC8796020

[19] Simon GE, Johnson E, Shortreed SM et al (2024) Predicting suicide death after emergency department visits with mental health or self-harm diagnoses. Gen Hosp Psychiatry 87:13–19. 10.1016/j.genhosppsych.2024.01.009 38277798 10.1016/j.genhosppsych.2024.01.009 PMC10939795

[20] Sheu Y-h, Simm J, Wang B, Lee H, Smoller JW (2025) Continuous time and dynamic suicide attempt risk prediction with neural ordinary differential equations. NPJ Digit Med 8(1):161. 10.1038/s41746-025-01552-y 40082653 10.1038/s41746-025-01552-y PMC11906764

[21] Bayramli I, Castro V, Barak-Corren Y et al (2021) Temporally informed random forests for suicide risk prediction. J Am Med Inform Assoc 29(1):62–71. 10.1093/jamia/ocab225 34725687 10.1093/jamia/ocab225 PMC8714280

[22] Simon GE, Shortreed SM, Boggs JM et al (2022) Accuracy of ICD-10-CM encounter diagnoses from health records for identifying self-harm events. J Am Med Inform Assoc 29(12):2023–2031. 10.1093/jamia/ocac144 36018725 10.1093/jamia/ocac144 PMC9667165

[23] Stewart C, Crawford PM, Simon GE (2017) Changes in coding of suicide attempts or self-harm with transition from ICD-9 to ICD-10. Psychiatr Serv 68(3):215–215. 10.1176/appi.ps.201600450 27903145 10.1176/appi.ps.201600450

[24] Simon GE, Ziebell RA, Johnson E, Shortreed SM Do health records data accurately identify repeat self-harm after emergency department visits? *medRxiv*. 2025:2025.06.26.25330358. 10.1101/2025.06.26.25330358

[25] Hedegaard H, Schoenbaum M, Claassen C, Crosby A, Holland K, Proescholdbell S (2018) Issues in developing a surveillance case definition for nonfatal suicide attempt and intentional self-harm using international classification of diseases, tenth revision, clinical modification (ICD-10-CM) coded data. Natl Health Stat Rep. Feb;(108):1–19 29616901

[26] Tsui FR, Shi L, Ruiz V et al (2021) Natural language processing and machine learning of electronic health records for prediction of first-time suicide attempts. JAMIA Open. 10.1093/jamiaopen/ooab011 33758800 10.1093/jamiaopen/ooab011 PMC7966858

[27] Bayramli I, Castro V, Barak-Corren Y et al (2021) Temporally informed random forests for suicide risk prediction. J Am Med Inform Assoc 28(1):62–71. 10.1093/jamia/ocab225 34725687 10.1093/jamia/ocab225 PMC8714280

[28] Wolock CJ, Williamson BD, Shortreed SM et al Importance of variables from different time frames for predicting self-harm using health system data. J Biomed Inform. 2024 Dec;160:104750. . 10.1016/j.jbi.2024.104750 10.1016/j.jbi.2024.104750 PMC11891787 39557209

[29] Doupnik SK, Rudd B, Schmutte T et al (2020) Association of suicide prevention interventions with subsequent suicide attempts, linkage to follow-up care, and depression symptoms for acute care settings: a systematic review and meta-analysis. JAMA Psychiatr 77(10):1021–1030. 10.1001/jamapsychiatry.2020.1586 10.1001/jamapsychiatry.2020.1586 PMC7301305 32584936

[30] Skopp NA, Smolenski DJ, Bush NE et al (2022) Caring contacts for suicide prevention: A systematic review and meta-analysis. Psychol Serv. 2023 Feb;20(1):74-83. 10.1037/ser0000645 10.1037/ser0000645 35420858

[31] Nuij C, van Ballegooijen W, de Beurs D et al (2021) Safety planning-type interventions for suicide prevention: meta-analysis. Br J Psychiatry 219(2):419–426. 10.1192/bjp.2021.50 35048835 10.1192/bjp.2021.50

[32] The Joint Commission. National patient safety goal of suicidal prevention. Accessed Aug 6 (2024) : https://www.jointcommission.org/standards/standard-faqs/behavioral-health/national-patient-safety-goals-npsg/000002241/

[33] Brown LA, Boudreaux ED, Arias SA et al (2020) C-SSRS performance in emergency department patients at high risk for suicide. Suicide Life Threat Behav 50(6):1097–1104. 10.1111/sltb.12657 32706437 10.1111/sltb.12657 PMC7746629

[34] McCabe R, Bergen C, Lomas M, Ryan M, Albert R (2023) Asking about self-harm during risk assessment in psychosocial assessments in the emergency department: questions that facilitate and deter disclosure of self-harm. BJPsych Open 9(3):e93e93. 10.1192/bjo.2023.32 37226481 10.1192/bjo.2023.32 PMC10228282

[35] Bergen C, Bortolotti L, Temple RK et al (2023) Implying implausibility and undermining versus accepting peoples’ experiences of suicidal ideation and self-harm in emergency department psychosocial assessments. Original research. Frontiers Psychiatry 2023-August-30 14. 10.3389/fpsyt.2023.1197512 10.3389/fpsyt.2023.1197512 PMC10499316 37711424

[36] Ellison AG, Jansen LAW, Nguyen F et al (2022) Specialty psychiatric services in US emergency departments and general hospitals: results from a nationwide survey. Mayo Clin Proc 97(5):862–870. 10.1016/j.mayocp.2021.10.025 35410751 10.1016/j.mayocp.2021.10.025

[37] Boudreaux ED, Larkin C, Camargo CA, Miller IW (2020) Validation of a secondary screener for suicide risk: results from the emergency department safety assessment and follow-up evaluation (ED-SAFE). Jt Comm J Qual Patient Saf. 10.1016/j.jcjq.2020.03.008 10.1016/j.jcjq.2020.03.008 PMC7276296 32417230

[38] Boudreaux ED, Larkin C, Kini N et al (2018) Predictive utility of an emergency department decision support tool in patients with active suicidal ideation. Psychol Serv 15(3):270–278. 10.1037/ser0000236 30080084 10.1037/ser0000236 PMC6082179

[39] Oquendo MA, Wall M, Wang S, Olfson M, Blanco C (2024) Lifetime suicide attempts in otherwise psychiatrically healthy individuals. JAMA Psychiatr 81(6):572–578. 10.1001/jamapsychiatry.2023.5672 10.1001/jamapsychiatry.2023.5672 PMC10882500 38381442

[40] Sveticic J, Stapelberg NC, Turner K (2020) Suicidal and self-harm presentations to emergency departments: the challenges of identification through diagnostic codes and presenting complaints. Health Inf Manag J 49(1):38–46. 10.1177/1833358319857188 10.1177/1833358319857188 31272232

[41] Swain RS, Taylor LG, Braver ER, Liu W, Pinheiro SP, Mosholder AD (2019) A systematic review of validated suicide outcome classification in observational studies. Int J Epidemiol 48(5):1636–1649. 10.1093/ije/dyz038 30907424 10.1093/ije/dyz038

[42] Huang X, Ribeiro JD, Franklin JC (2020) The differences between individuals engaging in nonsuicidal self-injury and suicide attempt are complex (vs. complicated or simple). Front Psychiatry. 10.3389/fpsyt.2020.00239 32317991 10.3389/fpsyt.2020.00239 PMC7154073

[43] Chartrand H, Tefft B, Sareen J et al (2022) A longitudinal study of correlates, discharge disposition, and rate of re-presentation to emergency services of adults who engage in non-suicidal self-injury. Arch Suicide Res 26(3):1141–1158. 10.1080/13811118.2020.1856259 33306000 10.1080/13811118.2020.1856259

